# Inhibition of Eph receptor A4 by 2,5-dimethylpyrrolyl benzoic acid suppresses human pancreatic cancer growing orthotopically in nude mice

**DOI:** 10.18632/oncotarget.5729

**Published:** 2015-10-19

**Authors:** Hironobu Takano, Toru Nakamura, Takahiro Tsuchikawa, Toshihiro Kushibiki, Kouji Hontani, Kazuho Inoko, Mizuna Takahashi, Shoki Sato, Hirotake Abe, Shintaro Takeuchi, Nagato Sato, Kei Hiraoka, Hiroshi Nishihara, Toshiaki Shichinohe, Satoshi Hirano

**Affiliations:** ^1^ Department of Gastroenterological Surgery II, Hokkaido University Graduate School of Medicine, Sapporo 060-8638, Japan; ^2^ Department of Translational Pathology, Hokkaido University Graduate School of Medicine, Sapporo 060-8638, Japan

**Keywords:** EphA4, prognostic factor, 2,5-dimethylpyrrolyl benzoic acid, human, pancreatic cancer, orthotopic models

## Abstract

Ephrin receptor A4 (EphA4) is overexpressed in human pancreatic adenocarcinoma (PDAC) and activate cell growth. Recent studies have identified small molecules that block EphA4. In this study, we investigated the correlation between EphA4 expression and the prognosis of patients with PDAC. We also examined the cytostatic efficacy of 2,5-dimethylpyrrolyl benzoic acid (compound 1), a small molecule that blocks EphA4, in PDAC cells. Overall survival of patients with EphA4 positivity was significantly shorter than that of patients with EphA4 negativity (*P* = 0.029). In addition, multivariate analysis revealed that EphA4 expression was an independent prognostic factor in PDAC patients (*P* = 0.039). Compound 1 showed a cytostatic efficacy in PDAC cells expressing EphA4 *in vitro* and *in vivo*. Our study indicated that compound 1 suppressed both EphA4 and Akt phosphorylations, and induced apoptosis in PDAC cells expressing EphA4. In conclusion,compound 1 has a high potential as a therapeutic agent for patients with PDAC.

## INTRODUCTION

Human pancreatic ductal adenocarcinoma (PDAC) is one of the most aggressive cancers, with a very poor prognosis [1, 2]. A recent study of cancer incidence and mortality has indicated that PDAC will become the second leading cause of cancer-related death by 2030 in the United States [3]. Surgical resection is the only curative treatment for PDAC. However, because of the difficulty to detect PDAC at an early stage, only 20% of patients with PDAC can be treated surgically [4]. Several treatments including chemotherapy and radiotherapy have been adopted for PDAC, but its prognosis continues to be insufficient to cure patients [5–7]. Therefore, new therapeutic strategies are needed to improve the prognosis of PDAC patients.

Previously, we analyzed the gene expression profiles of 18 pancreatic tumors and 29 normal human tissues, and identified hundreds of upregulated genes in pancreatic cancer [8, 9]. Another study has shown that Eph receptor A4 (EphA4) is overexpressed in PDAC and promotes cancer cell growth [10], suggesting that EphA4 is a likely prognostic factor or a molecular target for therapy of PDAC.

EphA4 is a member of the erythropoietin-producing hepatocellular (Eph) family of receptor tyrosine kinases. Eph receptors were first identified in the late 1980s [11]. The Eph family of receptors is the largest subgroup of receptor tyrosine kinases and is activated by interacting with cell surface ligands called ephrins. Eph receptors are classified into two subfamilies, type A and B, according to their binding affinities for ephrin ligands that are classified into two subclasses, glycosylphosphatidylinositol anchor (A type) and transmembrane domain (B type) [12]. In general, EphA receptors bind ephrin A ligands, but there are some exceptions including EphA4 binding ephrin B ligands. The roles of Eph receptors/ephrins have been reported in humans [13]. Eph receptor-ligand interactions result in bidirectional signals; a forward signal that depends on Eph kinase activity and a reverse signal that depends on Src family kinases [14]. Eph receptors and ligands have exhibited various expression patterns and roles in many types of human cancer cells [14, 15]. It is known that ephrin-dependent Eph signaling plays a role in cancer cell growth, migration, and invasiveness through various signaling pathways [15], including the Ras-Erk/Akt pathway, integrin-mediated adhesion, and epithelial-mesenchymal transition [16–19]. High expression of EphA4 is associated with a poor prognosis for human gastric and breast cancers [20, 21]. On the other hand, EphA4 expression is associated with improved outcomes in patients with lung adenocarcinoma [22]. A previous study showed that expression of EphA4 was not correlated with the prognosis of PDAC [23]. In fact, the roles of EphA4, including the related signaling pathways in several cancers, have not been studied extensively.

Noberini et al. identified a small molecule, 2,5-dimethylpyrrolyl benzoic acid (compound 1), that selectively inhibits binding of ephrin to EphA4 and Eph receptor A2 (EphA2) [24]. This finding has led to studies of Eph receptor/ephrin blockade as a strategy in anti-cancer therapy. Several short peptides and small molecules have been examined for interference of Eph receptor/ephrin binding [25, 26]. The efficacy of these peptides and molecules has been studied in diseases of the nervous system [27]. In a previous study, EphA4 expression was correlated with the prognosis of patients with amyotrophic lateral sclerosis (ALS), and blocking EphA4 with compound 1 increased the survival of ALS models *in vivo* [28]. Therefore, we hypothesized that compound 1 may have a therapeutic efficacy in PDAC, because EphA4 is upregulated in patients with PDAC.

In this study, we evaluated the utility of EphA4 as a biomarker to predict the survival of PDAC patients, and the possibility of EphA4 as a therapeutic target in PDAC using compound 1.

## RESULTS

### EphA4 and EphA2 expression in human PDAC tissues and its correlation with prognosis and clinicopathological factors

**Figure 1:**
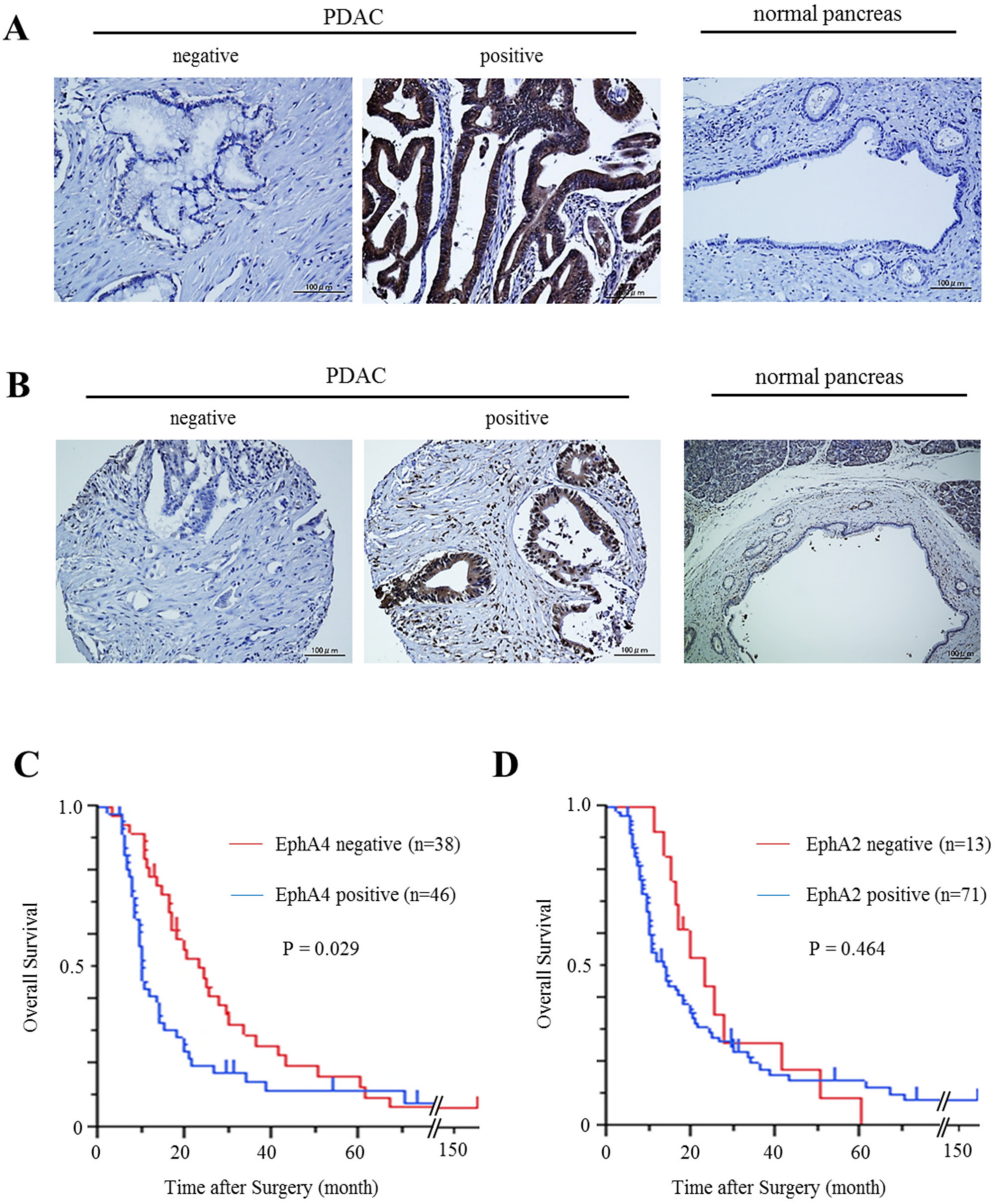
Expression of EphA4 and EphA2 in human PDAC tissues and its correlation with overall survival **A.** Immunohistochemical staining of EphA4 in human PDAC tissues and normal pancreatic tissues. Scale bar: 100 μm. **B.** Immunohistochemical staining of EphA2 in human PDAC tissues and normal pancreatic tissues. Scale bar: 100 μm. **C.** Kaplan-Meier survival analysis of overall survival for 84 resected PDAC samples and EphA4 expression. The EphA4-positive group showed significantly lower survival rates (*P* = 0.029). **D.** Kaplan-Meier survival analysis of overall survival for 84 resected PDAC samples and EphA2 expression. EphA2 was not correlated with the survival rates of PDAC patients (*P* = 0.464).

We examined the expression patterns of EphA4 and EphA2 in human PDAC tissues by immunohistochemical staining. There were EphA4- and EphA2-positive cases of human PDAC samples, but normal pancreatic duct tissues of all controls did not express EphA4 or EphA2 (Figure 1A and 1B). Among the 99 patients, expression of EphA4 and EphA2 was observed in the PDAC tissues of 46 (46.5%) and 71 (71.7%) patients, respectively. The median follow-up time of all patients was 14.1 months. Patients with EphA4 expression had significantly lower survival rates than those without EphA4 expression (*P* = 0.029, Figure 1C). In addition, the median survival times of patients with EphA4 positivity or negativity were 9.6 and 20.1 months, respectively. EphA2 expression was not correlated with the prognosis of PDAC patients (*P* = 0.464, Figure 1D). Next, we analyzed the relationship between the expression of EphA4 and clinicopathological factors in PDAC patients. As a result, EphA4 expression was not correlated with other factors (Supplementary Table S2). However, the expression of EphA4 was significantly associated with poorer overall survival in univariate analysis (HR 1.678; 95% CI 1.048–2.704; *P* = 0.030, Table 1). Moreover, similar to lymph node metastasis, univariate analysis indicated that EphA4 expression was an independent poor prognostic factor for PDAC patients (HR 1.648; 95% CI 1.025–2.667; *P* = 0.039, Table 1).

**Table 1:** Survival analysis of patients with PDAC

	*N* = 84	Univariate Analysis		Multivariate Analysis	
		HR (95% CI)	*P* value	HR (95% CI)	*P* value
Age (<70 / ≥ 70)	57 / 27	1.217 (0.732–1.973)	0.438		
Gender (M / F)	51 / 33	0.834 (0.509–1.341)	0.457		
Location (head / body, tail)	63 / 21	0.700 (0.387–1.195)	0.197		
CEA (<5.0 / ≥ 5.0)	43 / 41	1.540 (0.952–2.485)	0.077		
CA19-9 (<37.0 / ≥ 37.0)	19 / 65	1.595 (0.924–2.932)	0.095		
Tumor size (<2.5 / ≥ 2.5)	19 / 65	1.413 (0.821–2.572)	0.218		
EphA4 (−/ +)	38 / 46	1.678 (1.048–2.704)	0.030	1.648 (1.025–2.667)	0.039
Lymph node metastasis (−/+)	31 / 53	2.123 (1.273–3.652)	0.003	1.909 (1.136–3.310)	0.014
Lymphatic invasion (−/+)	22 / 62	1.438 (0.851–2.548)	0.178		
Venous invasion (−/+)	11 /73	2.129 (1.031–5.171)	0.040	1.979 (0.945–4.862)	0.072
Neural invasion (−/+)	7 / 77	1.830(0.851–4.771)	0.129		

### Expression of EphA4 and EphA2 in human PDAC cell lines

**Figure 2:**
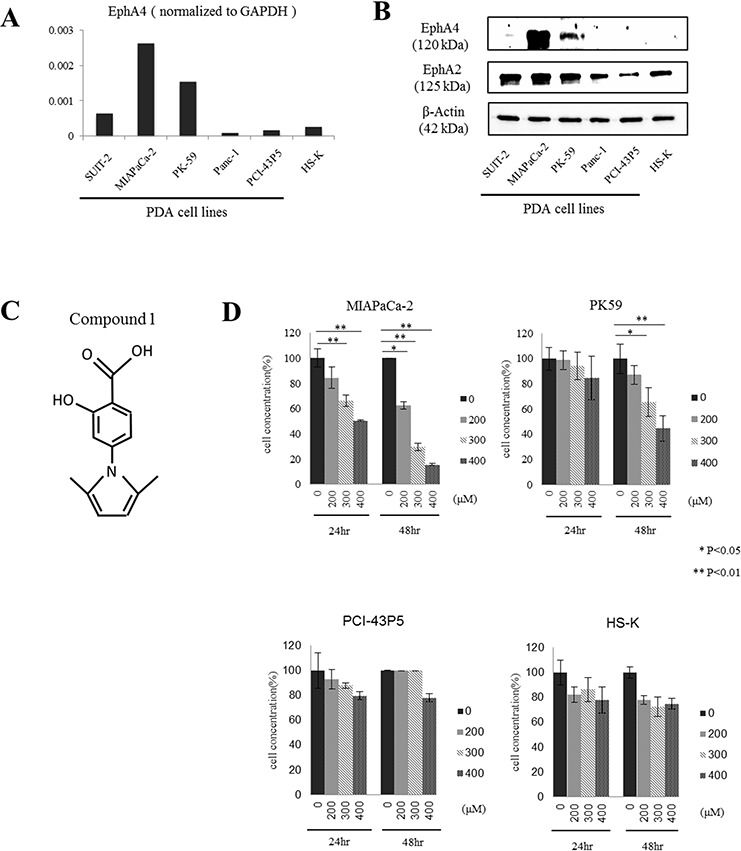
Expression of EphA4 and EphA2 in human PDAC cell lines and the effect of compound 1 on tumor cell proliferation *in vitro* **A.** Quantitative RT-PCR analysis of EphA4 expression in various PDAC cell lines. EphA4 expression was normalized to GAPDH. Data is shown as the mean of duplicate examinations. **B.** EphA4 and EphA2 expression in various PDAC cell lines and a normal diploid fibroblast cell line was assessed by western blotting. **C.** Structure of compound 1. The molecular weight is 231. **D.** Cell proliferation was assessed at 24 and 48 hours after compound 1 (200, 300, and 400 μM) treatment of MIAPaCa-2, PK-59, PCI-43P5, and HS-K cells by WST assays. Each experiment was performed three times. *P < 0.05; **P < 0.01.

Quantitative RT-PCR analysis showed high expression of EphA4 in MIAPaCa-2 cells and PK-59 cells, however, low expression of EphA4 in other PDAC cell lines or the human normal diploid fibroblast cell line HS-K (Figure 2A). Western blotting showed same results of EphA4 expression, while expression of EphA2 was observed in all cell lines including HS-K (Figure 2B).

### Blocking EphA4 with compound 1 inhibits the proliferation of PDAC cells

Next, we examined cytostatic effects by blocking EphA4 and EphA2 with compound 1 in PDAC cells using a WST assay (Figure 2C). EphA4-positive MIAPaCa-2 cells and PK-59 cells, EphA4-negative PCI-43P5 cells, and HS-K cells as a normal control were used in this experiment. Compound 1 showed cytostatic effects in MIAPaCa-2 cells or PK-59 cells, and no effects in PCI-43P5 or HS-K cells (Figure 2D). At 24 hours after application of compound 1, the proliferation of MIAPaCa-2 cells was significantly inhibited by more than 300 μM compound 1 compared with 1% DMSO only (*P* < 0.01, Figure 2D). At 48 hours after application of compound 1, the proliferation of MIAPaCa-2 cells was significantly inhibited by more than 200 μM compound 1 compared with 1% DMSO only (*P* < 0.05 or 0.01, Figure 2D). The same result was found in PK-59 cells (Figure 2D), while the effect was weaker than MIAPaCa-2 cells. However, the proliferation of PCI-43P5 and HS-K cells was slightly inhibited by 400 μM compound 1. These results indicated that compound 1 exerted cytostatic effect in EphA4-positive cells in a concentration and time-dependent manner. And also the extent of cell growth inhibitory effect was consistent with the degree of expression of EphA4.

### EphA4 is associated with the Akt pathway in PDAC

**Figure 3:**
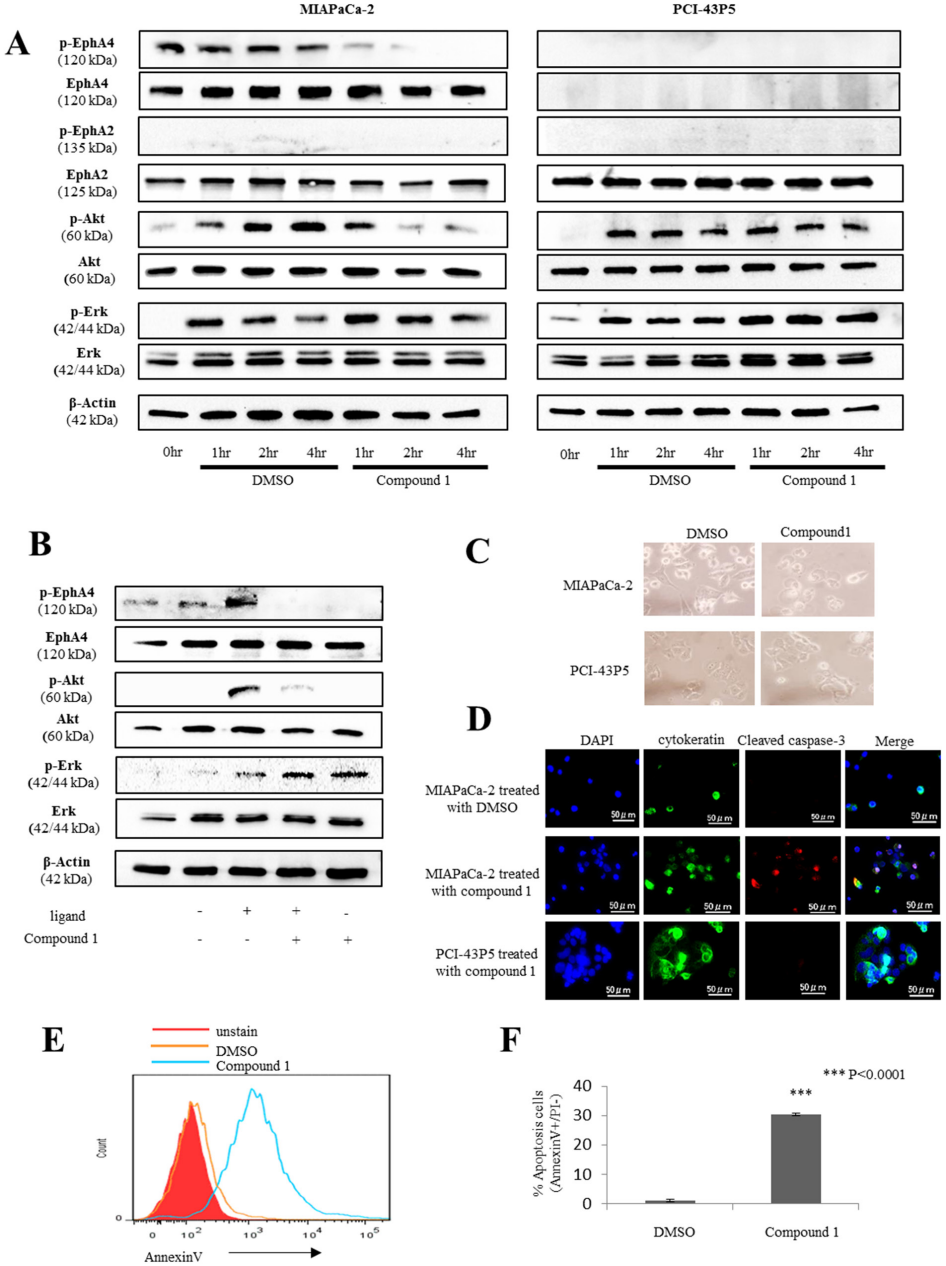
Compound 1 affects the Erk/Akt pathway and induces apoptosis in PDAC cells expressing EphA4 **A.** Western blotting of phosphorylated EphA4, EphA4, phosphorylated EphA2, EphA2, phosphorylated Akt, total Akt, phosphorylated Erk, total Erk, and β-actin in 400 μM compound 1– or 1% DMSO only-treated MIAPaCa-2 and PCI-43P5 cells. **B.** Western blotting of phosphorylated EphA4, EphA4, phosphorylated Akt, total Akt, phosphorylated Erk, total Erk, and β-actin in 10 ng/ml ligand and/or 400 μM compound 1- treated MIAPaCa-2. **C.** Representative photomicrographs of MIAPaCa-2 and PCI-43P5 cells treated with 400 μM compound 1 or 1% DMSO only for 2 hours. **D.** MIAPaCa-2 cells treated with 400 μM compound 1 or 1% DMSO only for 2 hours and PCI-43P5 cells treated with 400 μM compound 1 for 2 hours stained for cytokeratin (green) and cleaved caspase-3 (red) by immunofluorescence with nuclear counterstaining (DAPI; blue). Scale bar: 50 μm. **E.** Flow cytometric analysis of annexin V in MIAPaCa-2 cells treated with 400 μM compound 1 or 1% DMSO only for 2 hours. Red histogram: unstain of annexin V; Orange line: 1% DMSO only; Green line: compound 1. Representative data are shown. **F.** Percentage of early apoptotic cells (annexin V positive and PI negative) in 1% DMSO only- and compound 1-treated groups. The experiment was performed three times. ***P < 0.0001.

We investigated the signaling pathways associated with EphA4 in PDAC by blocking EphA4 with compound 1. We focused on two signaling pathways activated in cancer, Akt and Erk pathways, because a previous report showed that Akt and Erk pathways are correlated with cell proliferation as a downstream pathway of Eph/ephrin interactions [15]. First, we found that EphA4 phosphorylation was suppressed by 400 μM compound 1 (Figure 3A). Next, we found that Akt phosphorylation was suppressed at 2 and 4 hours after application of 400 μM compound 1 in MIAPaCa-2 cells (Figure 3A). Moreover, Erk phosphorylation was slightly increased by compound 1 in MIAPaCa-2 cells (Figure 3A). In PCI-43P5 cells, both Akt and Erk pathways were unchanged by treatment with compound 1 or 1% DMSO only (Figure 3A). These results indicated that compound 1 inhibited EphA4 and Akt phosphorylation only in EphA4-positive cells. Under administration of ligand of EphA4, EphA4 phosphorylation and Akt phosphorylation were also suppressed by 400 μM compound 1 (Figure 3B). And, Erk phosphorylation was increased by compound 1 (Figure 3B). It was indicated that compound 1 blocked binding between ligand and EphA4 and suppressed EphA4-Akt pathway.

### Compound 1 induces apoptosis in EphA4-positive PDAC cells

MIAPaCa-2 and PCI-43P5 cells were treated with 400 μM compound 1. After 2 hours of treatment, a change in cell morphology was only observed in MIAPaCa-2 cells (Figure 3C). Next, we evaluated apoptosis in PDAC cells treated with compound 1. Immunofluorescence staining of MIAPaCa-2 cells treated with 400 μM compound 1 for 2 hours showed cleaved caspase-3 that is activated in apoptotic cells (Figure 3D). Flow cytometry showed a larger number of annexin V-positive cells among MIAPaCa-2 cells treated with compound 1 compared with the control (Figure 3E). In addition, the rate of early apoptotic cells, which were defined as annexin V positive and PI negative, was significantly higher in MIAPaCa-2 cells treated with compound 1 than in the control (*P* < 0.0001, Figure 3F). On the other hand, in PCI-43P5 cells, the rate of early apoptotic cells in each group was not significantly different (*P* = 0.062, Supplementary Figure S1). These data indicated that compound 1 induced apoptosis only in EphA4-positive PDAC cells.

### Validation of live imaging using MIAPaCa-2 cells expressing firefly luciferase

**Figure 4:**
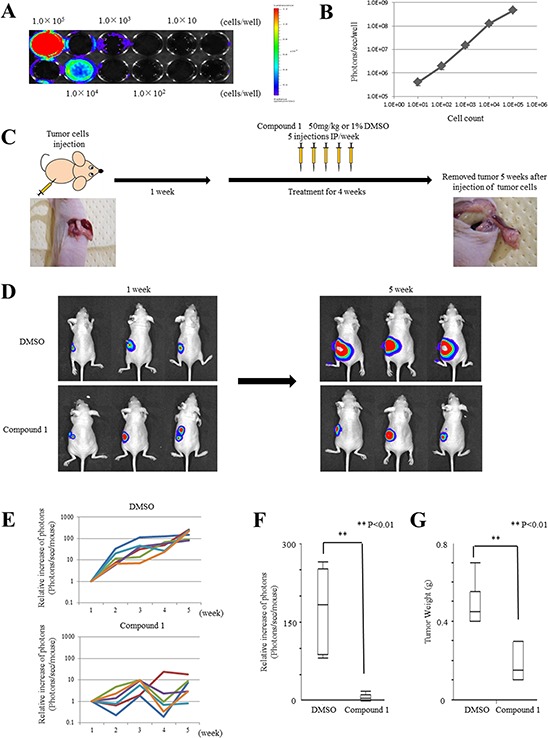
Compound 1 suppresses cancer cell growth in orthotopic mouse models **A.** *In vitro* imaging of luciferase-expressing MIAPaCa-2 cells. Cells were seeded at 1 × 10^1^, 10^2^, 10^3^, 10^4^, and 10^5^ per well. A representative image is shown. **B.** Relationship between the cell number and luminescent signal intensity in MIAPaCa-2.Fluc cells. The experiment was performed three times. **C.** Therapy schedule for orthotopic mouse models. **D.** Representative examples of bioluminescence imaging after tumor cell injection at 1 week and 5 weeks (4 weeks after initiation of therapy). Three cases are shown for each group (1% DMSO only- and compound 1-treated groups). **E.** Mice were analyzed by optical bioluminescence imaging at 1, 2, 3, 4, and 5 weeks after tumor cell injection. Treatment with 1% DMSO only or compound 1 was started at 1 week after tumor cell injection. The graph shows the signal intensities recorded from individual mice for each week and group. The signal intensity at 1 week was set at 1 as a control for each mouse. Each group had six mice. **F.** Relative signal intensity at 5 weeks compared with 1 week after tumor cell injection in each group. Data are presented as the mean ± SEM (1% DMSO only-treated group; *N* = 6, compound 1-treated group; *N* = 6). **P < 0.01. **G.** Tumor weights at 5 weeks after tumor cell injection in each group. Data are presented as the mean ± SEM (1% DMSO only-treated group; *N* = 6, compound 1-treated group; *N* = 6). **P < 0.01.

To assess the therapeutic effect of compound 1 in individual animals using an orthotopic PDAC mouse model, we first prepared MIAPaCa-2 cells transduced with a conventional replication-defective lentiviral vector carrying the firefly luciferase gene (MIAPaCa-2.Fluc). Stable expression of luciferase in these cells was confirmed by optical imaging *in vitro*, which showed a quantitative correlation between the MIAPaCa-2.Fluc cell number and luminescent signal intensity (Figure 4A and 4B).

### Compound 1 suppresses tumor growth in an orthotopic PDAC mouse model

We tested the therapeutic efficacy of compound 1 *in vivo* using the orthotopic mouse model and MIAPaCa-2.Fluc cells. The tumor cells were injected into the pancreas of Balb/c-nu/nu mice as described in the Materials and Methods. Prior to the first therapeutic injection at 1 week after tumor cell implantation, we checked for the presence of growing orthotopic tumors by optical imaging. Tumors were detected and mainly confined to the pancreas (Figure 4D). Next, the therapy was initiated following the schedule described in the Materials and Methods (Figure 4C). Six mice each were administered with compound 1 or 1% DMSO only (control). The mice were analyzed by optical imaging on days 7, 14, 21, 28 and 35 after tumor cell implantation. Quantitative analysis at various time points was performed by measuring photons/sec/mouse and expressed as the relative increase of photons compared with day 7 (Figure 4E). The results of three representative cases in each group on days 7 and 35 after tumor cell implantation are shown in Figure 4D. All mice in the control group showed tumor progression as evidenced by increasing bioluminescent signal intensities. However, mice in the compound 1-treated group showed minor tumor progression compared with the control group. On day 35 after tumor cell implantation, mice in the compound 1-treated group showed significant signal inhibition compared with the control group (*P* = 0.0039, Figure 4F). On day 35 after tumor cell implantation, mice in the compound 1-treated group had significantly lower tumor weights than the control group (*P* = 0.0033, Figure 4G). These results showed the efficacy of compound 1 to inhibit PDAC tumor growth by i.p administration.

### *In vivo* effects of compound 1 on orthotopic tumors and major organs

**Figure 5:**
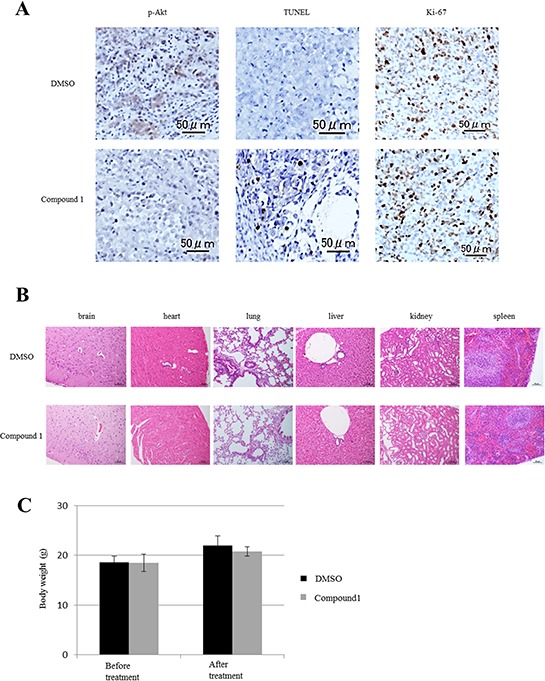
Effect of compound 1 on orthotopic tumors and major organs *in vivo* **A.** Immunohistochemical staining of phosphorylated Akt and ki-67 in orthotopic PDAC tumors *in vivo*. Apoptotic cells were analyzed by a TUNEL assay. A representative example is shown for compound 1- and 1% DMSO only-treated groups. Scale bar: 50 μm. **B.** Major organs at the conclusion of treatment were stained with hematoxylin and eosin. Scale bar: 50–200 μm. **C.** The body weight of mice at before treatment and after treatment in each group. Data are presented as the mean ± SEM (1% DMSO only-treated group; *N* = 6, compound 1-treated group; *N* = 6).

We analyzed the *in vivo* effects of compound 1 on orthotopic tumors and major organs in mouse models by immunohistochemical staining. First, as previously shown by *in vitro* assays (Figure 3), we investigated whether compound 1 suppressed Akt phosphorylation and induced apoptosis in orthotopic tumors. Akt phosphorylation levels were lower in the compound 1-treated group than in the control group (Figure 5A). More apoptotic cells were also found in the compound 1-treated group than in the control group by TUNEL assays (Figure 5A). The number of apoptotic cells in the compound 1-treated group was significantly larger than that in the control group (*P* = 0.0046, Supplementary Figure S2). The frequency of Ki-67-positive cells showed no significant difference between compound 1-treated and control groups (data not shown). Next, we investigated the side effects of compound 1 on major organs including the brain, heart, lung, liver, kidney, and spleen. These organs were collected after treatments and subjected to immunohistochemical staining and TUNEL assays. There were no differences in the brain tissue after treatment of both compound 1-treated and control groups (Figure 5B), such as structure difference or neuronal loss. And we could not find any neurological disorder during the treatment in mice. No significant findings were noted in the other major organs of both compound 1-treated and control groups (Figure 5B). In addition, TUNEL assays showed no apoptotic cells in the major organs of both groups (data not shown). And, the change of body weight of mice in each group during treatment was nearly equal (Figure 5C).

## DISCUSSION

Our study indicated that the expression of EphA4 was correlated with the overall survival of patients with PDAC. Furthermore, EphA4 was an independent prognostic factor in PDAC. Many retrospective analyses have reported clinicopathological variables, such as the tumor size, lymph node metastasis, and CA19-9 levels, which had a correlation with the survival of PDAC patients [29–31]. In these previous studies, lymph node metastasis was indicated as the strongest prognostic factor in PDAC [32]. Our results also showed that lymph node metastasis was the strongest prognostic factor, and EphA4 was the second most significant prognostic factor in PDAC compared with the other clinicopathological factors. It has been previously reported that high expression of EphA4 is correlated with the prognosis of patients with breast and gastric cancers [20, 21]. However, Giaginis et al. analyzed 67 PDAC patients and reported that only EphA5 and EphA7 were correlated with the patient prognosis [23]. In the data base which is COSMIC, EphA4 is mutated in 2 samples in all 251 samples of PDACs. Mutation type is substitution missense in both of 2 samples. It is considered that the mutation of EphA4 is not remotely related with PDACs because there are a quite few cases with mutation of EphA4 in PDACs. In this study, we analyzed more patients with PDAC and found that EphA4, but not EphA2, was correlated with the prognosis, suggesting that EphA4 is a useful prognostic biomarker for PDAC.

Pancreatic cancer is often resistant to chemotherapy or radiotherapy. To improve the prognosis of patients with pancreatic cancer, it is necessary to investigate new therapeutic targets or strategies. Because Nakamura et al. and Iiizumi et al. found that EphA4 was overexpressed in PDAC and related to the proliferation of cancer cells [9, 10], we focused on EphA4 as a new therapeutic target for PDAC. In 2008, Noberini, et al. reported a small molecule, termed compound 1, which selectively inhibits ephrin binding to EphA4 and EphA2 [24]. In this study, we found that compound 1 exerted a cytostatic effect on EphA4-positive PDAC cells only. EphA2 was expressed more widely in PDAC cell lines than EphA4, but compound 1 showed no cytostatic effect on EphA2-positive PDAC cells. EphA2 has been correlated with cancer cell survival, and some previous studies have reported that therapeutic targeting of EphA2 is effective for some cancers [25, 33, 34]. However, our results suggested that EphA2 had no major correlation with the survival of PDAC cells.

Next, we investigated the mechanism underlying the suppression of PDAC cell proliferation by compound 1. In our study, it was indicated that phosphorylation level of EphA4 in PDAC cells was upregulated. EphA4 was activated without administration of ligand. The fact indicated the possibility that EphA4 was activated by ligand which was expressed in PDAC cells themselves or EphA4 itself or other ligand-independent factors. In a previous study, it was found that the Erk/Akt pathway was activated in pancreatic cancer cells and played a role in pancreatic cancer cell proliferation [35]. Moreover, Eph receptors/ephrins are related to these pathways [36, 37]. Therefore, we focused these pathways and showed that compound 1 suppressed Akt phosphorylation and slightly activated Erk phosphorylation. Both Erk and Akt pathways are regulated by the small GTPase Ras [38]. However, the Akt pathway can be activated independently of Ras and there is cross-talk regulation between Erk and Akt pathways. Rommel, et al. demonstrated that Akt phosphorylation inhibits the Erk pathway [39], and Menges, et al. showed that activation of Erk signaling causes negative feedback inhibition of the Akt pathway [40]. Our results indicated that compound 1 suppressed the Akt pathway and upregulated the Erk pathway, which is consistent with these previous reports. And, under administration of ligand of EphA4, compound 1 also suppressed EphA4-Akt pathway. It showed that compound 1 had the suppressive effect on ligand-dependent EphA4 signaling pathway in PDAC cells. In addition, the present data showed that compound 1 induced apoptosis in PDAC cells via the Akt pathway. This pathway regulates various cellular functions including apoptosis [41]. Datta et al. found that Bad is a target gene of Akt, and that unphosphorylated Bad forms a heterodimer with Bcl-XL or Bcl-2 and blocks their anti-apoptotic activity [42], triggering an imbalance in the levels Bcl-2 family members and the release of cytochrome C. As a result, the caspase cascade including caspase-3 is induced and stimulates PARP cleavage, an indicator of apoptosis initiation [43]. We showed that compound 1 suppressed Akt phosphorylation and upregulated cleaved caspase-3 in PDAC cells, suggesting that compound 1 induced apoptosis via the Akt pathway in EphA4-positive PDAC cells.

*In vivo* examination showed that compound 1 suppressed tumor growth by i.p. administration. Furthermore, compound 1 suppressed Akt phosphorylation and induced apoptosis in EphA4-positive PDAC tumors. We also analyzed the expression of Ki-67 in orthotopic tumors. Ki-67 is regarded as a proliferation marker correlated with cell cycle progression [44]. However, we found no difference in the expression of Ki-67 between the treatment and control groups. In terms of the side effects of compound 1, we found no significant effects on major organs by compound 1. Therefore, compound 1 may be safe for use in therapy of patients with PDAC in the future.

In conclusion, compound 1 has great potential as a therapeutic agent for patients with PDAC.

## MATERIALS AND METHODS

### Human samples

For tissue microarray (TMA) analysis, 99 resected tissue samples were obtained from PDAC patients at our institute between 1999 and 2005. TMA blocks were constructed as described in our previous report [45]. To increase accuracy, the TMA was constructed with multifold redundancy (four spots for cancer tissue and three spots for normal pancreatic tissue from each patient). Patients without information on survival and clinicopathological factors were omitted from analysis. A total of 84 patients were subjected to the analysis. Informed consent was obtained from patients, and the study was approved by the Institutional Review Board of Hokkaido University Hospital (No. 014-0221).

### Cell lines and culture

Human PDAC cell lines (MIAPaCa-2, PK-59, and Panc-1) were obtained from the American Type Culture Collection (ATCC, Rockville, MD, USA). SUIT-2, which is a human PDAC cell line, and HS-K, which is a human cell line of normal diploid fibroblasts, were obtained from RIKEN (Tokyo, Japan). PCI-43P5, which is a human PDAC cell line, was previously established from surgically resected primary carcinoma tissues in our laboratory [46]. All cell lines were cultured in appropriate media (MIAPaCa-2 and Panc-1 in Dulbecco's modified Eagle's medium, HS-K in minimum essential medium α, and the other cell lines in Roswell Park Memorial Institute medium) containing 10% fetal bovine serum (Cell culture Bioscience, Tokyo, Japan) and 1% penicillin-streptomycin (Life Technologies, Tokyo, Japan) at 37°C with 5% CO_2_.

### Immunohistochemical staining

Paraffin-embedded specimens were cut into thin sections and mounted on glass slides. The sections were deparaffinized in xylene and rehydrated in ethanol. Antigen retrieval was performed by boiling for 15 minutes in citrate buffer (pH 6.0). Endogenous peroxidase activity was blocked with 0.3% hydrogen peroxide in methanol. Nonspecific reactions were blocked with 10% normal goat serum (Nichirei, Tokyo, Japan) and antibody diluent with background reducing components (DAKO, Tokyo, Japan). Immunohistochemical reactions were carried out using enzyme polymer methods with the Histofine series (Nichirei). Primary antibodies were applied to the sections overnight at 4°C followed by 20 minutes of incubation with secondary antibodies at room temperature. A list of primary antibodies is shown in Supplementary Table S1. Immunohistochemical reactions were visualized with DAB (Nichirei) followed by counterstaining with hematoxylin and mounting with coverslips. The intensity of EphA4 staining was evaluated using following criteria: strong positive (scored as 2+), dark brown staining in more than 50% of tumor cells; weak positive (1+), any lesser degree of brown staining appreciable in tumor cells; absent (scored as 0), no appreciable staining in tumor cells. Positive cases were accepted only as strongly positive (2+) staining if three reviewers independently defined them as such. For apoptosis analysis *in vivo*, we performed TUNEL assays using a commercially available kit (Apoptosis *in situ* Detection Kit; WAKO, Osaka, Japan) according to the manufacturer's instructions.

### Quantitative RT-PCR

Total RNAs were extracted from cells using an RNeasy Plus Mini Kit (QIAGEN, Tokyo, Japan) according to the manufacturer's protocol and used for cDNA synthesis (Prime Script RT Master Mix, TAKARA BIO, Shiga, Japan). cDNA products were used to amplify target genes using Power SYBR Green (Life Technologies). PCR reactions and data analysis were performed in a StepOne Real-time PCR system (Applied Biosystems, Tokyo, Japan), using the comparative C_T_ method and the housekeeping gene GAPDH. Primers used in this study are as follows: GAPDH (Forward: 5′-GAAGGTGAAGGTCGGAGTC-3′, Reverse: 5′- GAAGATGGTGATGGGATTTC-3′), EphA4 (Forward: 5′-GACCTGAAACTGTAGCAGTGACTC-3′, Reverse: 5′-TGCCAACGCTGCTCCTG-3′). Primers specificity was confirmed by peak melt curve before using. All experiments were performed in duplicate for each sample.

### Western blotting

Cell lysates were prepared using RIPA buffer containing protease inhibitors aprotinin and PMSF. Protein samples were resolved by 7.5% or 15% SDS-polyacrylamide gel electrophoresis and then transferred to nitrocellulose membranes (GE Healthcare, Little Chalfont, England). The membranes were probed with primary antibodies against target molecules (Supplementary Table S1) overnight at 4°C followed by incubation with secondary antibodies conjugated to horseradish peroxidase for 1 hour at room temperature. Secondary antibodies were purchased from Jackson ImmunoResearch. Immunoreactivity was detected by the Image Lab & ChemiDoc XRS Plus system (BIO RAD, Tokyo, Japan). β-Actin served as a loading control.

### Cell proliferation assay (WST assay)

Cell proliferation was assessed using a Cell Counting Kit-8 (DOJINDO, Kumamoto, Japan). MIAPaCa-2, PK-59, PCI-43P5, and HS-K cell lines were used in this assay. Cells (5 × 10^3^/well) were seeded in 96-well plates and cultured for 24 hours before treatment. The cells were then treated with 200, 300, and 400 μM compound 1 or 1% DMSO only for 24 and 48 hours. At 4 hours after addition of 10 μl WST-8 reagent to each well, absorbances were measured at 450 nm using a microtiter plate reader. The viability of cells treated with 1% DMSO only was set at 100%, and the absorbance of blank wells with medium and without cells was set at zero.

### Cell culture method in analysis of signaling pathway

Cells, MIAPaCa-2 and PCI-43P5, were applied in 6-well plate at 1 × 10^5^ cells and pre-incubated for 24 hours, following cultured with medium with 10% FBS and added 400 μM compound 1 or 1% DMSO only in each well and incubated for 1, 2, 4 hours. For investigating interaction between ephrin and EphA4, MIAPaCa-2 cells were applied in 6-well plate at 1 × 10^5^ cells and pre-incubated for 24 hours, following cultured with medium without FBS for 2 hours. After that, 400 μM compound 1 or 1% DMSO only were added in each well. At 1 hour after that, 10 ng/ml recombinant human ephrin-A4 (R&D system, Minneapolis, USA) or PBS only were added each well and incubated for 1 hour.

### Immunofluorescence

Cells were fixed with 4% paraformaldehyde for 30 minutes, permeabilized with 0.5% Triton X-100 for 5 minutes, and then blocked with 1% bovine serum albumin for 1 hour. Primary anti-pan-cytokeratin (Affymetrix, Tokyo, Japan) and anti-cleaved caspase-3 (Cell Signaling Technology, Tokyo, Japan) antibodies were added to the cells, followed by incubation for 1 hour at room temperature. After washing with PBS, the cells were incubated with Alexa Fluor 555 goat anti-rabbit IgG (Life Technologies) for 1 hour, and then mounted on slides using Dapi-Fluoromount-G (SouthernBiotech, Birmingham, USA). The cells were imaged with a BIOREVO BZ-9000 (Keyence, Osaka, Japan).

### Flow cytometry

Flow cytometry were conducted using an Annexin V-FITC Early Apoptosis Detection Kit (Cell Signaling Technology) according to the manufacturer's instructions. Cells were harvested by trypsinization and resuspended at a density of 1 × 10^6^ cells/ml in 1 × binding buffer. After double staining with FITC-Annexin V and propidium iodide (PI), the cells were analyzed using a FACS Canto II flow cytometer (BD Biosciences, Palo Alto, California, USA).

### Orthotopic pancreatic cancer models and therapy schedule

Female BALB/c-nu/nu mice were purchased from CLEA Japan. At 6 weeks of age, a left subcostal incision was made in the mice under anesthesia induced with ketamine (100 mg/kg) and xylazine (10 mg/kg). MIAPaCa-2 cells expressing luciferase (MIAPaCa-2.Fluc) were adjusted to 5 × 10^5^ cells/30 μl HBSS containing Matrigel (Corning, NY, USA) and injected below the capsule of the pancreas just beneath the spleen using a 27 G needle and 50 μl Hamilton syringe. The abdominal wound was closed by suturing with 3–0 non-absorbable surgical sutures. These procedures were carried out according to a previous report [47]. From 1 week after injection of the cancer cells, compound 1 (50 mg/kg, five times per week) or 1% DMSO only were intraperitoneally (i.p.) administered for 4 weeks. The mice were then sacrificed to evaluate tumor weights and perform immunohistochemical analysis of the tumors and major organs including the brain, lung, heart, liver, kidney, and spleen. Animal procedures were conducted according to the guidelines of the Hokkaido University Institutional Animal Care and Use Committee using an approved protocol.

### Optical imaging analysis

Bioluminescence in cultured cells or live mice was examined by optical imaging using a cooled CCD system (Xenogen IVIS). For *in vitro* imaging, luciferase expression of MIAPaCa-2.Fluc cells was confirmed at 10 minutes after addition of luciferin (10 μl/well; Promega, Tokyo, Japan). For *in vivo* imaging, mice were anesthetized with ketamine (100 mg/kg) and xylazine (10 mg/kg) and then i.p. administered luciferin (200 μl/mouse). After 10 minutes, bioluminescent signals were analyzed with the Xenogen IVIS with a 10 second exposure time. The mice were analyzed weekly from 1 week after injection of the cancer cells. To quantitate the tumor burden, bioluminescent signals were calculated from the imaging data using Living Image software 3.2 (Xenogen, Alameda, California, USA) according to the manufacturer's protocol.

### Statistical analysis

Unless indicated otherwise, the results are presented as the mean ± standard error of the mean of at least three independent experiments. Data were analyzed using the Mann-Whitney *U*-test, Fisher's exact test, or χ2 test as appropriate. Overall survival was calculated from the date of surgery to the date of the last follow-up or patient death. Univariate survival analysis was performed according to the Kaplan-Meier method. Survival differences were estimated by the log-rank test. Multivariate analysis was conducted using the Cox proportional hazards regression model. A *P*-value of less than 0.05 was considered significant. The confidence interval (CI) was determined at the 95% level. All data were analyzed using JMP® 10 software.

## SUPPLEMENTARY FIGURES AND TABLES


