# Supplementary material for: Inhibition of Eph receptor A4 by 2,5-dimethylpyrrolyl benzoic acid suppresses human pancreatic cancer growing orthotopically in nude mice
Source: Oncotarget. 2015 Oct 19;6(38):41063–76. doi: 10.18632/oncotarget.5729 (PMC4747390; doi:10.18632/oncotarget.5729)
Supplement: Supplementary file 1 [file oncotarget-06-41063-s001.pdf]

SUPPLEMENTARY FIGURES AND TABLES

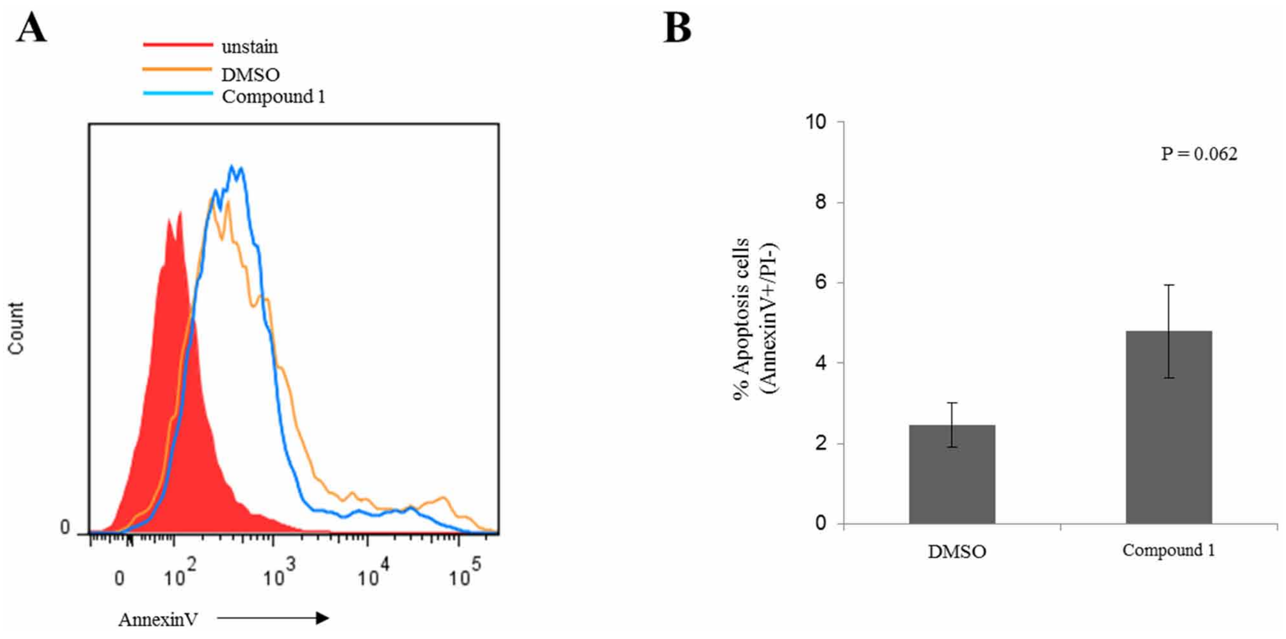

Supplementary Figure S1: Flow cytometric analysis of annexine V in PCI-43P5 cells. A. Flow cytometric analysis of annexin V in PCI-43P5 cells treated with 400 µM compound 1 or 1% DMSO only for 2 hours. Red histogram: unstain of annexin V; Orange line: 1% DMSO only; Green line: compound 1. Representative data are shown. **B.** Percentage of early apoptotic cells (annexin V positive and PI negative) in 1% DMSO only- and compound 1-treated groups. The experiment was performed three times. $P = 0.62$.

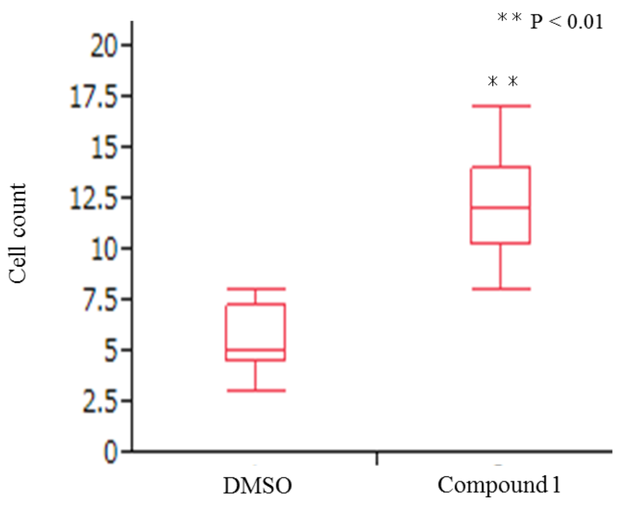

Supplementary Figure S2: Frequency of apoptotic cells in orthotopic tumors. Frequency of apoptotic cells in orthotopic tumors after treatment with compound 1 or 1% DMSO only. Cells were counted in three independent high-power (×200) microscopic fields for each sample. There were six cases in each group. $^{**}P < 0.01$.

Supplementary Table S1: List of antibodies used for immunohistochemical staining and western blotting

Immunohistochemical staining

Molecule	Clonality	Dilution	Manufacture
Eph receptor A4	Rabbit polyclonal	1 : 100	Abcam
Eph receptor A2	Rabbit polyclonal	1 : 50	Abcam
Ki-67	Mouse monoclonal	1 : 300	Dako
Phospho-Akt (Ser473)	Rabbit monoclonal	1 : 50	Cell Signaling

Western blotting

Molecule	Clonality	Dilution	Manufacture
EphA4 Receptor	Mouse monoclonal	1 : 150	Invitrogen
EphA2 (D4A2)	Rabbit monoclonal	1 : 1000	Cell Signaling
EphA4 phospho-specific (Tyr-602)	Rabbit polyclonal	1 : 500	ECM Biosciences
Phosphor-EphA2 (Tyr594)	Rabbit polyclonal	1 : 1000	Cell Signaling
p44/42 MAPK (Erk1/2) (137F5)	Rabbit monoclonal	1 : 1000	Cell Signaling
Phospho-p44/42 MAPK (Erk1/2) (Thr202/Try204)	Rabbit monoclonal	1 : 2000	Cell Signaling
Akt	Rabbit polyclonal	1 : 1000	Cell Signaling
Phospho-Akt (Ser473)	Rabbit polyclonal	1 : 1000	Cell Signaling
β- Actin (C4)	Mouse monoclonal	1 : 1000	Millipore

Supplementary Table S2: Association of EphA4 with clinicopathological findings

	EphA4 positive (*N* = 46)	EphA4 negative (*N* = 38)	*P*-value
Gender			0.630
male	29	22	
female	17	16	
Age			0.803
	68(44–89)	65(45–82)	
Location			0.076
Pancreas head	38	25	
Pancreas body/tail	8	13	
CEA			0.886
	5.85(1.3–39.7)	3.4(1.1–70.8)	
CA19–9			0.379
	170.25(1.0–6605.9)	116.7(1.0–19238.4)	
Tumor size			0.805
	3.2(1.0–7.3)	3.0(1.5–6.0)	
pStage(UICC)			0.388
2a ≤	15	16	
2b ≥	31	22	
Lymph node metastasis			0.388
negative	15	16	
positive	31	22	
Lymphatic invasion			0.981
negative	12	10	
positive	24	28	
Venous invasion			0.506
negative	5	6	
positive	41	32	
Neural invasion			0.509
negative	3	4	
positive	43	34	
